# Well-care visit attendance among children of adolescent mothers in South Africa: a theory-informed mixed-methods study

**DOI:** 10.1186/s12889-026-27326-z

**Published:** 2026-05-28

**Authors:** Camille Wittesaele, Hlokoma Mangqalaza, Elona Toska, Lucie Cluver, Helen A. Weiss, Aoife M. Doyle

**Affiliations:** 1 International Statistics and Epidemiology Group, Department of Infectious Disease Epidemiology, London School of Hygiene & Tropical Medicine, London, UK; 2 Centre for Social Science Research, University of Cape Town, Cape Town, South Africa; 3 Department of Social Policy & Intervention, University of Oxford, Oxford, UK; 4 Department of Public Health, Institute of Tropical Medicine Antwerp, Antwerpen, Belgium; 5 Department of Psychiatry and Mental Health, University of Cape Town, Cape Town, South Africa; 6 Biomedical Research and Training Institute in Zimbabwe, Harare, Zimbabwe

**Keywords:** Adolescent mothers, Child health records, Well-care visits, HIV-exposed infants, Health behaviour, South Africa, COM-B

## Abstract

**Background:**

WHO recommend that children and adolescents attend scheduled well-care visits for health promotion, prevention, early detection, identification and management of disability and disease. Children born to adolescent mothers experience a disproportionate burden of poor health outcomes, but their well-care attendance is under-researched.

**Methods:**

Cross-sectional data were used from a cohort of adolescent and young mothers (10–24 years; *n* = 1040) and their children (*n* = 1145) recruited through purposive, convenience-based sampling across healthcare facilities and community-based settings in Eastern Cape, South Africa (2017–2019). Quantitative data on visit attendance up to 18 months were analysed using descriptive statistics for children of adolescent mothers (10–19 years) aged ≥ 19 months at data collection, to allow complete observation of visits. In 2022, semi-structured interviews (*n* = 16) were conducted until saturation, to explore factors influencing attendance. Themes were developed and matched to the capability, opportunity and motivation model of behaviour (COM-B) and Theoretical Domains Framework using a realist thematic template analysis approach.

**Results:**

Records were available for 415/482 eligible children. Attendance declined from 85.1% (95%CI: 81.3–88.4) at 6 weeks to 49.7% (95%CI: 46.6–56.5%) at 18 months, with higher attendance during visits coinciding with the childhood immunisation schedule. Attendance and qualitative findings were similar by maternal HIV status. Themes were matched to the COM-B. *Capability*: mothers’ organisational and financial acumen facilitated their child’s attendance; mothers persisted in attending despite harsh attitudes from healthcare workers. *Opportunity*: financial and kinship support and information in the child health booklet facilitated visit attendance; lack of childcare support, poor weather, cost and distance to clinic interfered with attendance. *Motivation*: mothers were motivated to attend visits to gain knowledge and fulfil their parental role.

**Conclusion:**

This study identified missed opportunities for promoting life-course health and well-being among children and their adolescent mothers, and theory-informed opportunities to support well-care visit attendance. Findings challenge deficit-based narratives highlighting mothers’ motivations and strategies for ensuring attendance. Enhancing the quality of nurse-adolescent mother interactions may increase the perceived and actual value of visits and support attendance. Further research is needed on interventions to promote consistent attendance, address barriers to access and identify opportunities to strengthen integration of well-care and HIV-related services.

**Supplementary Information:**

The online version contains supplementary material available at 10.1186/s12889-026-27326-z.

## Introduction

WHO guidance recommends that children and adolescents attend scheduled well-care visits for healthier life-course trajectories and intergenerational health and well-being [1]. Well-care visits are vital contact points for the early detection of health issues, monitoring the healthy growth and development of children and to reduce preventable mortality and morbidity. Despite the importance of well-care services, attendance at well-care visits is under-researched in South Africa, particularly among children of adolescent mothers. In low and middle-income countries, children born to adolescent mothers (10–19 years) are more vulnerable to infant mortality and morbidity than children of older mothers [2–4]. Children of adolescent mothers are more likely to be born preterm [5, 6], have increased risk of anthropometric failure (i.e., stunting, underweight, wasting) [2, 3, 7, 8], undernutrition [9], early breastfeeding cessation [7], vertical transmission of HIV [10–12] and delayed school progression [3].

In South Africa, well-care services are delivered for free at routine intervals in primary healthcare facilities and documented in government-issued home-based child health records called Road to Health (RtH) booklets [13] (see Table 1). All children are expected to attend scheduled visits for growth monitoring and promotion, childhood vaccination, deworming, vitamin A supplementation, tuberculosis screening, development screening and to ensure access to linked HIV services such as early infant HIV testing. Health promotion and prevention interventions delivered in the first 18 months are particularly important for long-term health and development [1]. Mothers also receive postnatal care and breastfeeding counselling during visits from birth to 6-months. Understanding well-care visit attendance is vital for informing strategies to support service uptake and provision, particularly for this vulnerable group at high risk of poor maternal and child outcomes in South Africa and the region.

In South Africa, adolescent birth rates are highest in rural provinces such as the Eastern Cape, where adolescent girls accounted for 17.1% of deliveries in 2020/21 [14]. Low access to health services, low or incorrect use of contraceptives and sexual risk behaviours drive early childbearing rates and HIV incidence among adolescent girls [15–18]. These behaviours persist into pregnancy leading to delayed and under-utilisation of antenatal services [8, 19] and lower rates of early postnatal follow-up [20]. Poor uptake of maternal health services among adolescent and young mothers (15–24 years) has been associated with lower uptake of childhood vaccinations [21, 22]. In the Eastern Cape province, where antenatal HIV prevalence exceeds 35%, prevention of mother-to-child transmission (PMTCT) programs are integrated with well-care services extending the reach of HIV-related care to children and their mothers [23]. Young maternal age is a predictor for disengagement in PMTCT services [11, 24, 25], driving risk of vertical HIV transmission among children of adolescent mothers and interfering with HIV care for mothers [10]. Therefore, well-care services are key to HIV service delivery and reaching adolescent girls and young women who account for a third of all new HIV infections in South Africa [26].

**Table 1:** Overview of well-care services and schedule up to 18 months in South Africa

Well-care services	3–6 days	6 wks	10 wks	14 wks	4 mths	5 mths	6 mths	7 mths	8 mths	9 mths	10 mths	11 mths	12 mths	14 mths	16 mths	18 mths
Growth monitoring	•	•	•	•	•	•	•	•	•	•	•	•	•	•	•	•
PMTCT/HIV	•	•	•				•						•			•
Tuberculosis screening				•	•	•	•	•	•	•	•	•	•	•	•	•
Immunisations^a^		•	•	•			•			•			•			•
Feeding	•	•	•	•	•	•	•									
Vitamin A							•						•			•
Deworming													•			•
Development screening				•			•			•						•
Oral health													•			

^a^In December 2015, the national Expanded Programme on Immunisation revised its schedule adding two additional vaccination visits at 6 and 12 months. Source: Road to Health Booklet (version issued in 2010)

Studies examining well-care services in South Africa have focused on coverage of individual services delivered at well-care visits (i.e., hearing screening, vitamin A supplementation, deworming and growth monitoring) [27–31] and barriers to implementation and integration of services [32–34]. Due to fragmentation of maternal, child and HIV services, we cannot rely on childhood vaccination or PMTCT coverage to estimate well-care visit attendance [29, 32, 33]. In sub-Saharan Africa, studies have examined coverage of vitamin A supplementation, deworming and growth monitoring [35, 36], integration of family planning with well-care services [37, 38] and caregiver perceptions and experiences of well-care services [39, 40]. In 2011, a health-facility study in Zambia including only children of adult mothers (*n* = 198), found that HIV-exposed children were more likely to receive well-care and non-HIV services than HIV-unexposed children [41]. While existing studies give us an understanding about barriers to delivering quality well-care services, there remains a lack of evidence about children’s attendance at these visits and the factors that influence their attendance. Maternal age is inconsistently reported in existing studies with no research examining its effect on attendance at well-care visits. This study examines birth to 18-month well-care visit attendance among children of adolescent mothers, including adolescent mothers living with HIV (AMLHIV), in the Eastern Cape, South Africa.

## Methods

### Study design

This mixed-methods study uses i) quantitative cross-sectional data from an observational cohort of adolescent and young mothers and their children, restricted to children born to adolescent mothers (10–19 years), and ii) qualitative data from semi-structured interviews (*n* = 16) with mothers from the cohort study conducted in 2022. The study adopted a sequential explanatory design, in which quantitative findings informed the qualitative interview guide to explore well-care visit attendance patterns identified in descriptive analyses [42]. Quantitative and qualitative findings are reported contiguously and interpreted narratively by weaving together insights from quantitative and qualitative results.

### Recruitment & sampling

#### Cohort recruitment and sampling for quantitative analysis

**Fig. 1:**
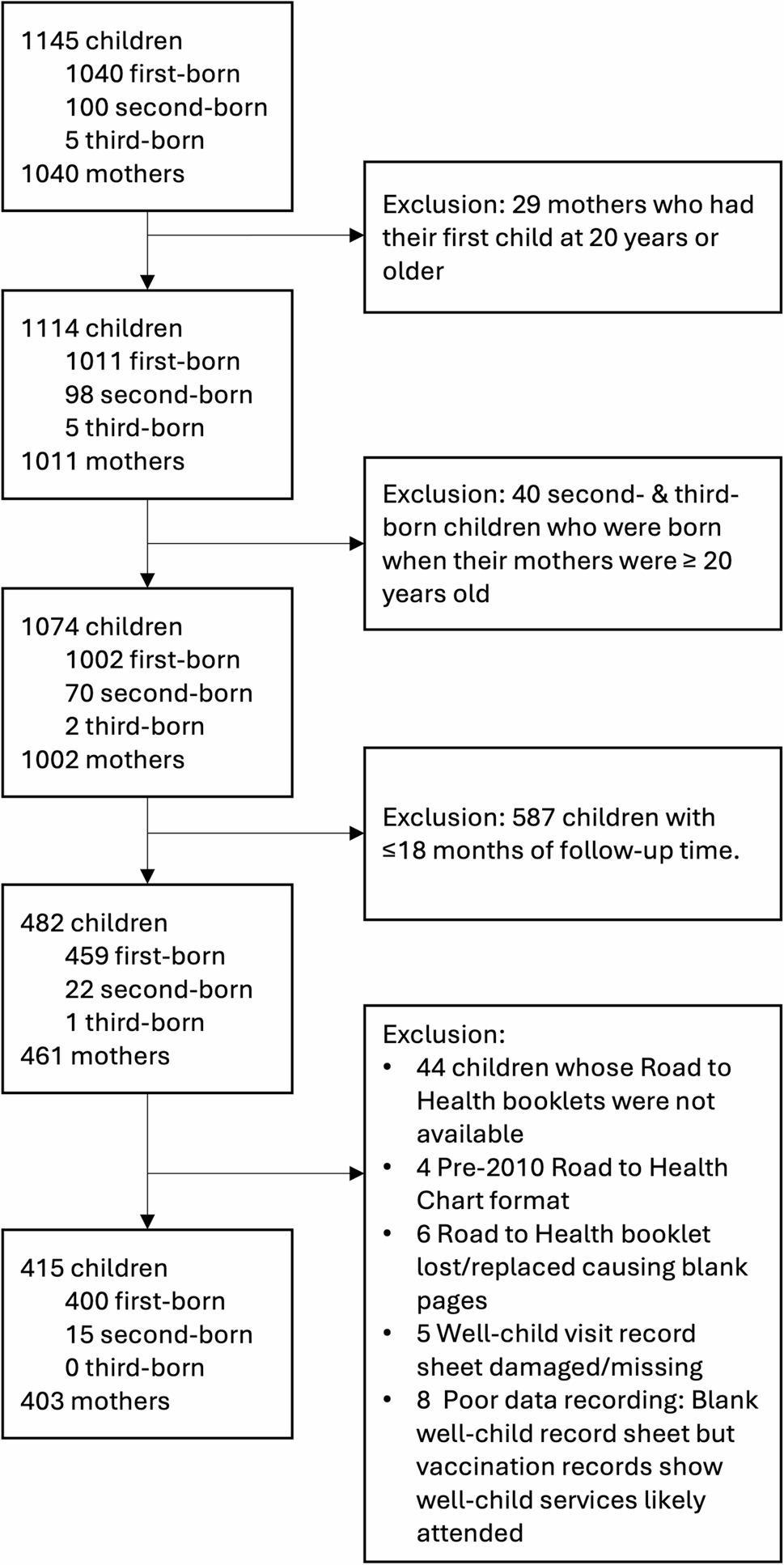
Study sample flowchart

The cohort included adolescent and young mothers (10–24 years) with at least one living child and were residing in an urban and rural health district of the Eastern Cape. All children born to eligible mothers were included regardless of age and cohabitation arrangements. To reduce selection bias towards health service users and improve representativeness across the district, multiple purposive and convenience-based recruitment strategies were implemented through health facilities and community-based settings [43]. These included all district health facilities, randomly selected secondary schools, and community-based channels (i.e., door-to-door recruitment, malls, salons, churches and social services). This approach enabled inclusion of children and adolescent mothers with varying levels of access to health services across the health district. As detailed in Fig. 1, data are only reported for children who were ≥ 19 months old at baseline data collection (2017-19) and had well-care visit attendance data available in their original RtH booklets (*n* = 415). This ensured a complete observation period for all visits from birth to 18 months, allowing sufficient time for the 18-month visit to have taken place and been recorded. Second and third order children were excluded if maternal age at birth was ≥ 20 years (*n* = 40). A further 67 eligible children were excluded due to: booklets were unavailable because they were either lost or not present at the interview location (*n* = 44), use of outdated child health card format in which well-care visit attendance was not recorded (*n* = 4), missing or damaged well-care visit record pages (*n* = 11) and poor-quality or incomplete well-care visit data (*n* = 8).

#### Qualitative study

In collaboration with quantitative researchers, mothers were purposively sampled from the main cohort and invited to participate in semi-structured qualitative interviews. A roster of participants in the cohort study was used to prioritise sampling of mothers of children ≤ 2 years old and mothers who were still ≤ 19 years old. This approach aimed to reduce recall time and bias among mothers who attended well-care visits further in the past. The sample included mothers who were selected from a range of characteristics including HIV status, number of children and age of their child(ren) as proxy for recent attendance at well-care visits. Interviews were conducted iteratively alongside data familiarisation and continued until no new topics or themes were identified (i.e., data saturation was reached) [44].

### Data collection procedures

All research tools were co-developed using a participatory approach during advisory group meetings and pilot interviews with adolescent mothers and an adolescent advisory group [45]. Researchers and health practitioners in adolescent and child health in South Africa were also consulted.

#### Quantitative

Adolescent mothers were recruited in their homes, following informed consent. Electronic survey forms were used to extract well-care visit attendance dates, date of immunisations and HIV-related information data from photos taken of RtH booklets. Data quality was monitored using a double-entry verification process, with independent extraction by trained research assistants, secondary validation, and random validation checks (5%) of extracted data by senior researchers. Socio-demographic and healthcare access factors were reported by mothers for each of their children. Where possible questionnaires used standardised measures designed for adolescents in South Africa (available at study’s website) [46]. Surveys were conducted using tablets with support from isiXhosa-speaking researchers. HIV status and dates of birth were validated using RtH booklets. Since maternal HIV status was recorded only at the time of data collection and may have been acquired after childbirth, it may not accurately reflect children’s exposure to HIV in utero.

#### Qualitative

Semi-structured interviews (maximum 60 min) were conducted telephonically due to COVID-19 pandemic-related precautions. Interviews were conducted in isiXhosa by a female interviewer (HM) with experience conducting qualitative research (including remotely) with adolescents and adolescent mothers, including those living with HIV. To support rapport-building and confidentiality, the interviewer was trained in remote interviewing, interviews were rescheduled when privacy or connection quality could not be assured, and preparatory reviews of RtH booklets were conducted to be familiar with each children’s well-care attendance, allowing for informed probing during interviews. After each interview, the interviewer completed a reflection form (Appendix 2). Adjustments to the interview guide were discussed during debrief meetings following every interview. A team of research assistants transcribed and translated all recorded interviews verbatim. As needed, unclear phrases were clarified with the interviewer and/or the transcriber. Notes gathered from reflection forms and debrief meetings provided contextual understanding and familiarity with the interview content, facilitating the identification of inconsistencies during transcript review. To further ensure accuracy and consistency in translation the interviewer also reviewed transcripts.

### Quantitative data

*Well-care visit attendance* was measured using binary categorisation (attended/ not attended) for each well-care visit up to 18 months. A visit was categorised as ‘attended’ if a well-care date was recorded alongside each visit on the well-care visit record page of the RtH booklet, and as ‘not attended’ where no date was recorded. Well-care visit attendance was measured as the proportion of children who attended each visit as a proportion of children ≥ 19 months (83 weeks old). Socio-demographic data (i.e., access to child support grants, residence, and children’s living arrangements) and healthcare access factors (i.e., distance to clinic in minutes and frequency of attendance at antenatal and postnatal care appointments) were collected from adolescent mothers using questionnaires.

### Qualitative data

#### Conceptual framework

**Fig. 2:**
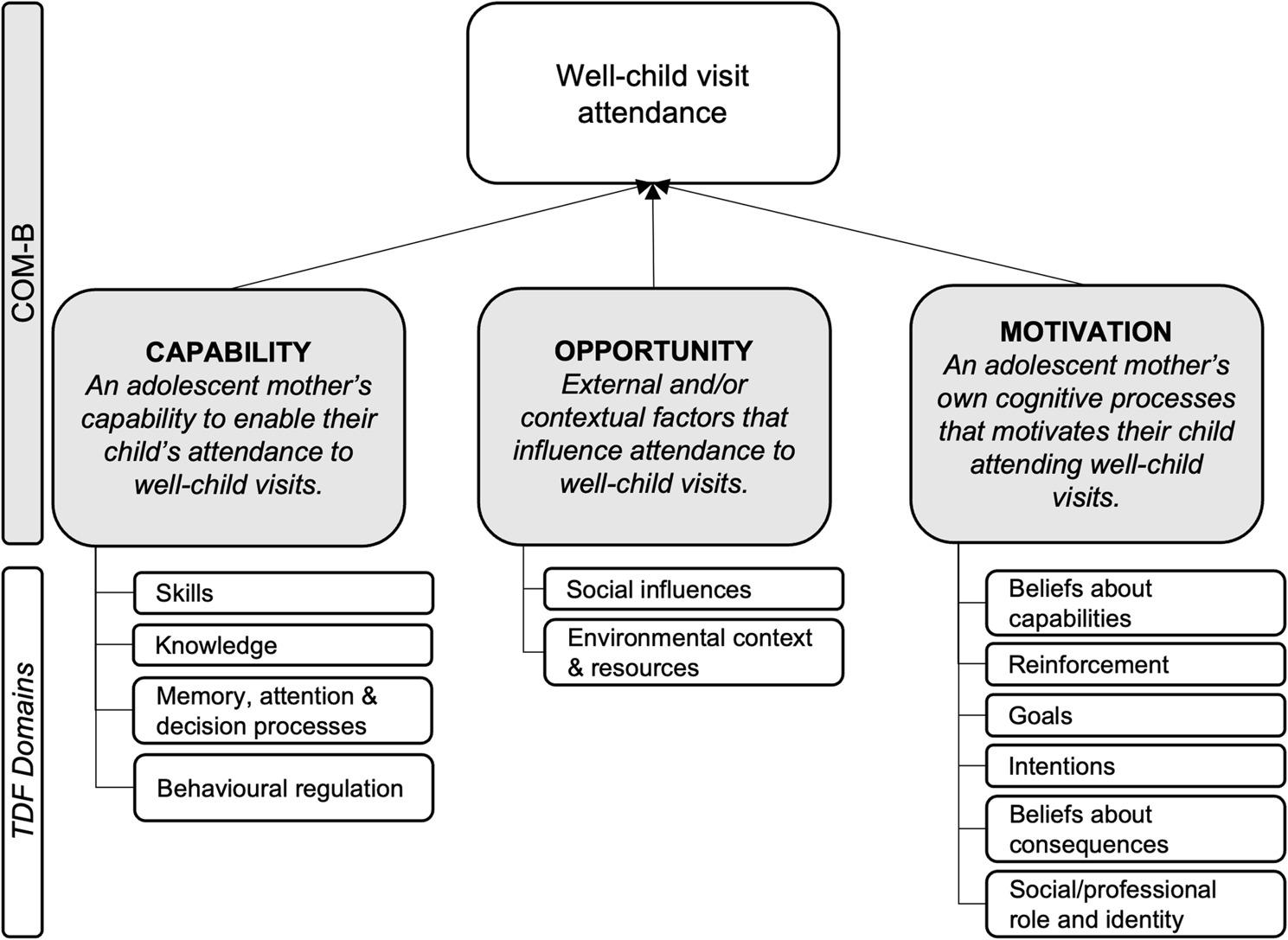
COM-B & TDF integrated framework for well-care visit attendance

This study applied a theory-informed approach using the Capability, Opportunity, Motivation model of Behaviour (COM-B) and the Theoretical Domains Framework (TDF) to explore well-care visit attendance behaviour among adolescent mothers [47–50] (see Fig. 2). The COM-B proposes three constructs which interact to produce behaviour. In this study, the *capability* construct encompasses an adolescent mother’s capability to enable their child’s attendance at well-care visits. The *opportunity* construct refers to external or contextual factors that influence attendance at well-care visits. The *motivation* construct covers an adolescent mother’s own cognitive processes that motivate the behaviour of interest. The TDF is integrated with the COM-B providing 14 domains to gain a deeper insight into barriers and facilitators to the behaviour of interest. Cane et al. have validated the TDF for use in health behaviour research and identified how TDF domains relate to each COM-B construct [47]. Fig. 2 presents the TDF domains mapped onto the COM-B which is used to examine well-care visit attendance behaviour among children of adolescent mothers [48, 49].

#### Interview guide

Interviews were conducted using a semi-structured interview guide (see Appendix 1) which was informed by the UNICEF Immunisation Caregiver Journey Framework [44] and broadly aimed to access narratives related to the constructs in the COM-B. Anchoring the interview guide to the three ‘high-level’ constructs in the COM-B enabled the interviewer to maximise interview time by concentrating on wider constructs that would generate narratives related to TDF domains. The temporal structure and questions in the Immunisation Caregiver Journey Framework were adapted to understand the lived experiences of adolescent mothers of children as they navigate well-care visits [51]. This included questions around decision-making and preparation for well-care visits, the journey to clinics and experiences during well-care visits. Further questions were included to ask about adolescent mothers’ knowledge of the well-care visit schedule, their use and understanding of the RtH booklets, timing and frequency of well-care visits, integration of HIV services and family planning services and whether they perceived any benefits for their own health. Vignettes were used to stimulate responses from participants and mitigate potential social desirability bias [52]. Short vignettes referred to hypothetical stories of adolescent mothers navigating well-care visits and HIV services including instances where their children may have skipped visits or delayed attendance. By normalising such experiences and shifting dialogue to the third person, this approach enabled us to solicit experiences from adolescent mothers and reduce response bias. The interview guide was translated with assistance from fieldwork research assistants using language designed to engage young people, back translated for accuracy, and piloted with participants from the cohort study.

### Data analysis

#### Quantitative

Sample characteristics report on sociodemographic and healthcare access characteristics by maternal HIV status. Outcomes are reported using descriptive statistics with 95% confidence intervals (CI). Differences by maternal HIV status were calculated using Pearson’s chi-squared test, Wilcoxon rank-sum test and t-test, as appropriate. Quantitative analyses were exploratory and aimed to characterise the cohort and patterns of well-care visit attendance rather than examining factors associated with attendance; analyses of factors associated with retention in the well-care visit schedule have been published separately [53]. Data were analysed using Stata/SE 17.0.

#### Qualitative

A realist thematic template analysis was conducted to identify mechanisms influencing well-care visit attendance [54]. First, two codebooks were generated: i) thematic codebook and ii) conceptual framework codebook [49]. The thematic codebook was generated using a deductive approach from the interview guide and literature. It was iteratively updated using an inductive approach based on data collection notes, debrief meetings after each interview and throughout familiarisation and coding. A conceptual framework codebook was generated using a deductive approach and included the 14 domains specified in the TDF. Interview transcripts were coded line-by-line by a single coder (CW) using NVivo, with codes grouped into potential themes and sub-themes. Analytic rigour was supported through iterative review and discussion with the interviewer. The lead researcher (CW) developed an initial thematic map based on themes [54], which was reviewed by the interviewer to assess the validity in relation to the data. This informed ongoing refinement, clustering and collapsing of codes. Discrepancies in interpretation were resolved through discussion. Finally, themes were reviewed, refined, and named, and then matched to domains in the conceptual framework in collaboration with the transcription team and the interviewer. This approach enabled us to agree on the meaning and interpretation of themes as well as match themes to domains in the framework. This study is reported in line with the Strengthening the Reporting of Observational Studies in Epidemiology (STROBE) checklist and the Consolidated criteria for reporting for qualitative research (COREQ) [55, 56].

## Results

Table 2 presents the sample characteristics of the 415 of 482 children aged ≥ 19 months included in the quantitative analysis (see Fig. 1). The median age of children at data collection was 2.7 years [interquartile range (IQR) 2.0-3.6] and the median age of mothers at the birth of their first child was 16.9 years [IQR 15.8–18.1]. Most children were singletons (79.8%; *n* = 331), with 71.8% (*n* = 298) living in an urban location and 76.2% (*n* = 307) living within 30 min travel time to a clinic. The majority (91.3%; *n* = 379) were cohabiting with their biological mother at least 4 nights per week and were reported to have ever had access to the child support grant (87.4%; *n* = 362). Over a third (39.0%; *n* = 157) of children’s mothers were living with HIV at the time of data collection and approximately 70.8% (*n* = 283) of mothers had progressed to secondary school at the time of their first birth. Attendance at antenatal services was high, with 68.0% of adolescent mothers (*n* = 282) attending 5 or more antenatal care appointments during their pregnancies. However, only 64.5% of mothers (*n* = 264) reported attending at least one postnatal care visit after each pregnancy.

**Table 2:** Sample characteristics of children and their adolescent mothers by maternal HIV status

Children (*n* = 415)	Total		Children of AMLHIV^a^ (*n* = 166)	Children of HIV-negative adolescent mothers (*n* = 249)	
	Frequency (%)				*p*-value
Child sex					
Female	207 (49.9)		80 (48.2)	127 (51.0)	0.6
Male	208 (50.1)		86 (51.8)	122 (49.0)	
Sibling					
Only child	331 (79.8)		114 (68.7)	217 (87.1)	< 0.001
Sibling	84 (20.2)		52 (31.3)	32 (12.9)	
Child age (years, median [IQR])					
	2.7 [ 2.0-3.6]		3.0 [2.3-4.0]	2.5 [2.0-3.3]	< 0.001
Access to child support grant					
Ever	362 (87.4)		150 (90.4)	212 (85.5)	0.1
Never	53 (12.6)				
Location					
Urban	298 (71.8)		126 (75.9)	172 (69.1)	0.1
Rural	117 (28.2)		40 (24.1)	77 (30.9)	
Child lives with biological mother					
≥ 4 nights/week	379 (91.3)		154 (92.8)	225 (90.4)	0.5
≤ 3 nights/week	9 (2.2)		4 (2.4)	5 (2.0)	
None	27 (6.5)		8 (4.8)	19 (7.6)	
Child distance to clinic					
≤ 20 min	181 (43.6)		76 (45.8)	105 (42.2)	0.4
21–30 min	126 (30.4)		54 (32.5)	72 (28.9)	
31–59 min	32 (7.7)		10 (6.0)	22 (8.8)	
≥ 60 min	64 (15.4)		21 (12.7)	43 (17.3)	
Unknown	12 (6.6)		5 (6.6)	7 (6.7)	

Mother (*n* = 403)	Total		AMLHIV^c^ (*n* = 157)	HIV-negative adolescent mothers (*n* = 246)	
Maternal age (years, median [IQR])^b^					
	16.9 [15.8–18.1]		17.8 [16.4–19.0]	16.6 [15.5–17.3]	< 0.001
Maternal education level at first pregnancy^d^					
Primary (≤ 8)	102 (25.5)		42 (26.9)	60 (24.6)	0.4
Secondary (9–12)		283 (70.8)	102 (65.4)	181 (74.2)	
Unknown		15 (3.8)	12 (7.7)	3 (1.2)	
Antenatal care appointments attended^e^					
≥ 5	282 (68.0)		108 (65.1)	174 (69.9)	0.3
≤ 4	57 (13.7)		16 (9.6)	41 (16.5)	
None	11 (2.7)		4 (2.4)	7 (2.8)	
Unknown	65 (15.7)		38 (22.9)	27 (10.8)	
Postnatal care appointments attended^f^					
≥ 1	264 (64.5)		96 (60.0)	168 (67.5)	0.1
None	145 (35.5)		64 (40.0)	81 (32.5)	

^a﻿^Unconfirmed if these children were HIV-exposed at birth as adolescent mothers may have acquired HIV after child’s birth

^b﻿^Maternal age at birth of first child

^c﻿^Maternal HIV status at the time of data collection

^d﻿^School grade when pregnant with first child

^e﻿^Number of antenatal visits attended during pregnancy for all children

^f^Number of postnatal visits attended for all children

Overall, AMLHIV (39.0%; *n* = 157) and their children (40.0%; *n* = 166) were older than HIV-negative adolescent mothers (61.0%; *n* = 246) and their children (60.0%; *n* = 249). Children of AMLHIV were also more likely to have a sibling. Maternal age at birth of first child was older among AMLHIV (17.8 years [IQR 16.4–19.0] versus 16.6 years [IQR 15.5–17.3]). The median age of children of AMLHIV was also higher than children of HIV-negative adolescent mothers (3.0 years [IQR 2.3-4.0] versus 2.5 years [IQR 2.0-3.3]). There were no other notable differences in characteristics by maternal HIV status.

### Well-care visit attendance

**Fig. 3:**
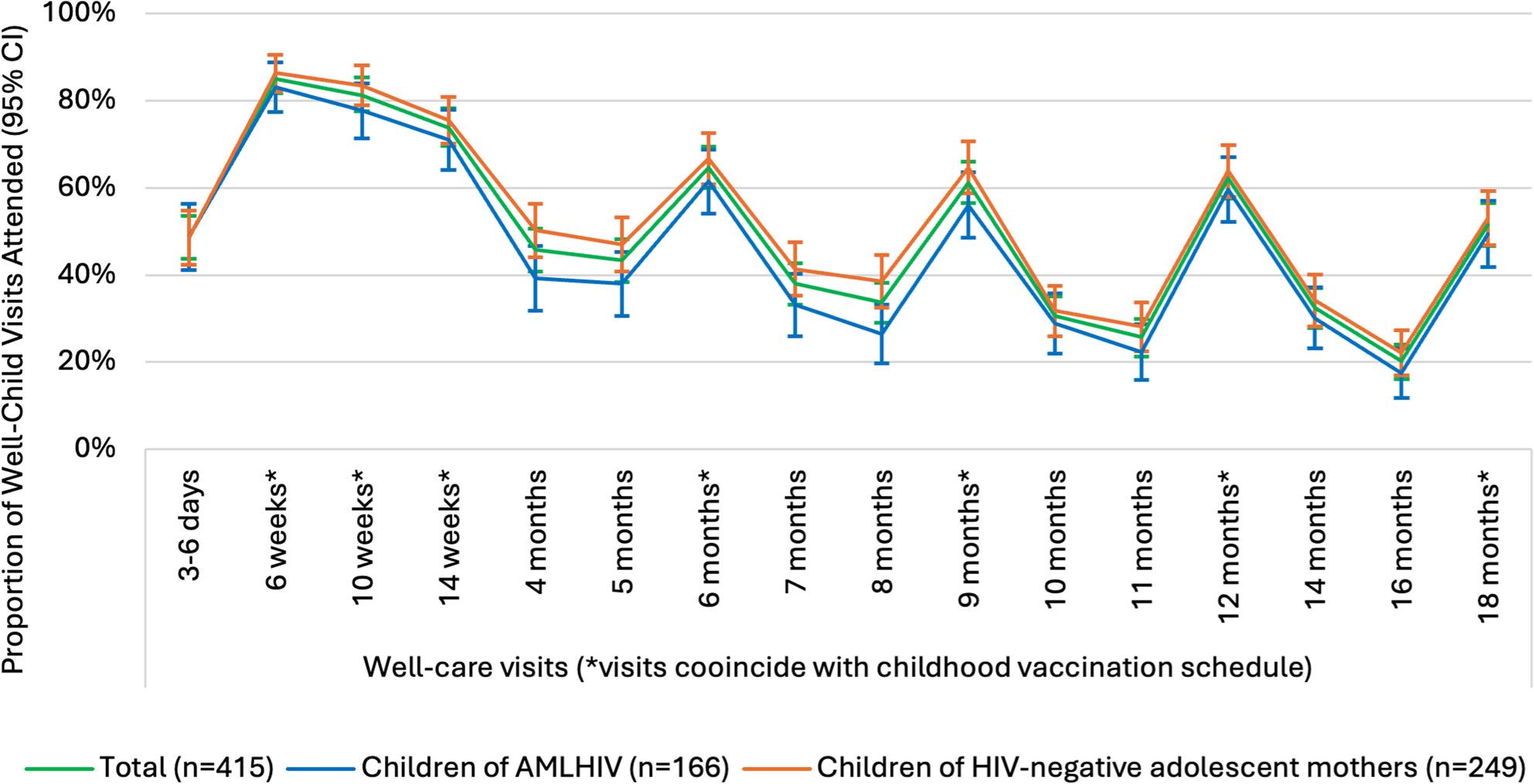
Proportion of children who attended well-care visits among children ≥19 months (*n*=415) by maternal HIV status. Note: Unconfirmed if children of AMLHIV were HIV-exposed at birth as adolescent mothers may have acquired HIV after child’s birth

The proportion of children who attended well-care visits up to 18 months (Fig. 3) was measured among children ≥ 19 months (*n* = 415) (see Supplement 1). Attendance at the first recommended well-care visit (3–6 days after birth) was the lowest, with only 48.7% of children having a recorded visit (95% CI: 43.8–53.6). The proportion of children who attended well-care visits was higher at the next visit but then gradually declined over time, from 85.1% (95%CI: 81.3–88.4) at 6 weeks, to 51.6% (95%CI: 46.6–56.5) at 18 months. Fig. 3 shows that a lower proportion of children of AMLHIV attended well-care visits from 6-weeks to 18-months compared to children of HIV-negative adolescent mothers. Unadjusted analyses found weak and mixed evidence that children of AMLHIV had lower attendance than children of HIV-negative adolescent mothers at 4 months (*p*-value: 0.03), 5 months (*p*-value: 0.07), 7 months (*p*-value: 0.09), 8 months (p-value: <0.01) and 9 months (p-value: 0.08). There was insufficient evidence indicating lower attendance among children of AMLHIV at other time points in the schedule (p-value: >0.10). A pattern emerged showing increased attendance during visits that coincided with the childhood immunisation schedule at 6–14 weeks, and 6, 9, 12 and 18 months respectively.

### Findings from semi-structured interviews

Sixteen mothers (18–27 years old at time interview; mean 17 years old at birth of first child) participated in semi-structured interviews lasting approximately 30–60 min (see Supplement 2 for participant characteristics). At the time of interview, four mothers were not living with their children, three were primiparous, seven were living with HIV and their children ranged between 8 months and 12 years old. Mothers used various modes of transport to reach well-care services and often received childcare support from their child’s aunt and grandmother. Twenty-one themes were developed, named and matched to domains in the TDF in collaboration with the interviewer and transcription team. No themes were identified for the ‘emotions’ and ‘optimism’ domain. The complete list of themes is summarised in Table 3. Nine major themes were selected which are described below and are organised according to the COM-B. While both inductive and deductive coding approaches were used, most themes reported in Table 3 were developed inductively, with deductive coding supporting the allocation of themes to relevant domains of the conceptual framework. 

#### Capability

i. Indifference & tolerance of harsh attitudes from nurses (TDF: behavioural regulation)

Mothers described being treated harshly by nurses and were disappointed about their approach to delivering health services. This was unsurprising to mothers who conveyed a tolerance towards these experiences. Mothers disregarded and excused harsh treatment from nurses so long as their children received services during well-care visits. Mothers were willing to tolerate harsh treatment since they ultimately benefited from information and guidance given by nurses.

*Yes*,* they shout*,* I have that thing that says there is a reason they shout. They would never just shout at you; they have a right… They are helping your life at the end… Without knowing them I love them; I love it maybe it’s being used to it. (#011: 24-year-old mother living with HIV; 2 children)*

ii. Compliance with nurse’s schedule (TDF: memory, attention, & decision processes)

Nurses and mothers used their children’s RtH booklets to track visits and attendance. Decision-making around the frequency and timing of well-care visits was determined by visit dates indicated by nurses in the RtH booklet. Mothers used this date to determine when they should next take their child for a well-care visit. In doing so, some mothers obeyed the nurses’ directions in respect to their authority.

*When you leave the clinic*,* you’re told that there’s a date when you’ll have to bring your child for vaccinations*,* and then on that date you must go to the clinic… They ask why you didn’t bring the child on his date. But*,* there’s nothing much. If you don’t bring him on his date*,* they do reprimand you though. They say*,* “why didn’t you bring your child on his date” and whatnot. (#010: 23-year-old mother living with HIV; 2 children)*

Despite their intention to comply with nurses’ instructions, some mothers decided to deviate from the well-care visit schedule. For example, two mothers self-determined which well-care visits they would attend by only taking their child for visits for vaccinations.

*There are some that I skip. I just get him checked when he’s going for his vaccinations. But I’m supposed to go every month but now it’s less. (#010: 23-year-old mother living with HIV; 2 children)*

However, overall visits were less likely to be entirely missed by choice and more likely to be delayed due to external factors (i.e., school commitments, lack of transport money, poor weather). There was an intention to attend well-care visits as close as possible to the date indicated by nurses.

*The challenge that I usually have*,* sometimes I do not take her for reasons being there is no transport money… So*,* I may not take her now*,* but take her in month-end… It would not be the day for me to take her to the clinic but take her anyway because she is supposed to go*,* and I am shouted at the clinic. (#011: 24-year-old mother living with HIV; 2 children)*

Well-care visits were not perceived to be an optional health service that one may deliberately choose to skip. Nurses monitored attendance using the RtH booklets and penalised mothers for missing well-care visits by reprimanding them verbally and sending them to the back of the queue.

*No*,* at the clinic they don’t allow you [to miss visits]*,* they say you’re last to whoever got there first. You’ll be last*,* even if someone came in after you*,* you’ll be the last one. Know that you’ll sit forever at the clinic. (#002: 22-year-old mother; 3 children)*

iii. Organisational & financial acumen (TDF: Skills)

Attending well-care visits required organisational skills and financial planning. High decision-making autonomy was conveyed as mothers were individually responsible for ensuring they had the resources (i.e., transport money and time) to take their child to the clinic and had childcare support for other children.

*I look at the child card and keep it*,* or write it on the phone as a reminder*,* and set the reminder. (#004: 20-year-old mother; 2 children)*

Constrained financial resources were maximised by combining trips to the clinic for multiple children in the household. Meanwhile, other mothers timed visits to the clinic around the payment schedule of government child support grants. If circumstances prevented mothers from escorting their children to well-care visits themselves, they endeavoured to make alternative arrangements to ensure their child’s attendance at the clinic. Children were often taken to well-care visits on behalf of the mother, by their child’s grandmother or aunt. This required foresight and coordination skills.

*[My sister] helped me to go [to the clinic] with [my child and hers] so that she can mind the other child. (#005: 20-year-old mother; 2 children)*

*[My mother] usually takes [my child] by herself if I am not around or at work. (#011: 24-year-old mother living with HIV; 2 children)*

#### Opportunity

iv. Barriers: Barriers to access, lack of childcare support, service delivery factors, COVID-19, weather-related (TDF: environmental context & resources)

Several barriers were described which interfered and delayed well-care visit attendance including (1) service-delivery factors such as clinic hours, unsuitable waiting area at clinic and interrupted delivery of services during COVID-19, (2) barriers to access such as distance to clinic and lack of transport money, (3) weather-related factors and (4) no-one being available to escort child to clinic.

*I have not gone to the clinic because … there is no waiting area that is proper there. If it is raining*,* the rain will be pouring on you and the baby outside… When there was COVID-19*,* I barely went… we were told even by the clinic that they are not going to open*,* they are going to fumigate and so on. (#008: 23-year-old mother living with HIV; 3 children)*

v. Facilitators: RtH booklet, responsive support, mother’s availability, accessibility of services (TDF: environmental context & resources)

RtH booklets were used as guides by mothers throughout the well-care visit schedule. Booklets were used to track the schedule and provided knowledge to help new mothers nurse their children when they were ill and to be able to identify symptoms which should prompt them to seek medical attention.

*Since you’ve never had a child*,* the clinic shows you how you take care of a child*,* what are the dos and don’ts when you have a child. (#001: 26-year-old mother; 2 children)*

Childcare and monetary support were leveraged from other caregivers to facilitate clinic attendance. When needed, other caregivers would escort children to well-care visits. As such, the availability of mothers did not inhibit children’s clinic attendance.

*It’s the support I was speaking about [that helps me attend the clinic]. [My child’s dad] knows first before I go*,* so we are able to prepare for all of that… so that’s why there are no challenges… He knows he has to give me money to go to the clinic. (#001: 26-year-old mother; 2 children)*

Mothers rarely received support that responded to their unique circumstances. For example, only one mother recalled a nurse scheduling the next well-care visit date for a date that was suitable for that mother’s schedule. Another mother’s school provided her with a letter to help her get prioritised in the well-care visit queue. This enabled her to return to school swiftly after taking her child to a well-care visit. While these instances improved the ability of these mothers to attend well-care visits on time, they were outliers.

*I struggle but because I have my teacher’s number*,* I ask an adult to call and explain what the problem is… I get a letter from school that explains that I am a student and I needed to bring my child to the clinic*,* so I’m served faster because of that. (#016: 18-year-old mother; 1 child)*

#### Motivation

vi. Opportunity to learn & reassurance about child’s health (TDF: Beliefs about capabilities)

Nurses were perceived to be a source of information among mothers. Mothers recalled feeling overwhelmed by their lack of knowledge about how to care for their infant. Consequently, they were motivated to attend well-care visits to access information and learn about how to care for their children.

*I learned a lot by taking my child for immunisation because it’s then explained to the parents in a way that she must breastfeed the child and PrEP*,* condom… you learn a lot. (#006: 23-year-old mother; 2 children)*

While mothers valued the opportunity to obtain information during these visits, few mothers felt that their own health and well-being benefited from attending visits. Mothers inconsistently recalled examples of nurses asking about their health post-birth. The perception of potential benefits to maternal health diminished over time as visits became primarily focused on their children’s health.

*It is for [my child’s] health*,* to check everything about her… I will say it is to check if she is well in her health. (#008: 23-year-old mother living with HIV; 3 children)*

Mothers felt that nurses were not adequately giving attention to their health and well-being and believed that nurses were only concerned about their children. This was identified as a desired area for improvement by one mother.

*Nurses only focus on the child’s health. That is what is important to them. They take care of the children very well*,* but I so wish that they could concentrate on us too as mothers and not only concentrate on children. (#014: 21-year-old mother; 2 children)*

Routine visits provided reassurance that their children were healthy and growing well.

*A nurse or doctor checks their [RtH] book and explains that he’s alright and healthy. (#016: 18-year-old mother; 1 child)*

Meanwhile, mothers tended to access health services for themselves (e.g., family planning and HIV-related services) during separate health visits.

vii. Fear of child illness if visits missed (TDF: Beliefs about consequences)

A perception that well-care visits are necessary for children to be healthy led to fears that children may become infected with vaccine-preventable diseases or that a child’s healthy growth and development could be hindered if visits were missed. Mothers routinely brought their children to well-care visits to ease these concerns. These fears may have been instigated by negative experiences accessing health services for ill children compared to routine well-care services. Negative experiences seeking curative health services were characterised by receiving incomplete services, being refused care, and being spoken to harshly by nurses. These may also have prompted mothers to attend well-care visits to avoid the possibility of needing to attend the clinic because their child was ill.

*I go to them*,* and they are important*,* I am not supposed to miss them because a baby is able to catch something while young… If I miss an immunisation*,* then she becomes sick and at the clinic they will say “you did not take her for immunisation maybe that is the reason you see those things”. (#008: 23-year-old mother living with HIV; 3 children)*

viii. Importance of well-care services & childhood immunisations (TDF: Intentions)

Mothers demonstrated high motivation to attend well-care visits. The importance of well-care services was prominent among mothers and for those who care for them and their children.

*It was alright*,* but it was difficult because you dread the distance*,* but you understand that it’s necessary. (#002: 25-year-old mother; 2 children)*

*This vaccine is necessary*,* even if I see him growing*,* he must get this injection…Your child should attend his vaccination appointments properly. (#012: 23-year-old mother living with HIV; 2 children)*

Mothers described instances of using their student financial aid and forfeiting school or work commitments to ensure that their child attended well-care visits.

*I give [my mother] money from my NSFAS [National Student Financial Aid Scheme]. We usually meet in town… I give [my mother] money a day before or the days before [the well-care visit]. (#004: 22-year-old mother; 3 children)*

Other caregivers also valued well-care visits. Mothers described multiple forms of support from their caregivers and the child’s father that facilitated clinic attendance. Family members provided cash and in-kind support (e.g., childcare support for other children to enable clinic attendance) to facilitate timely attendance at well-care visits.

*My sister takes him [to the clinic] because I am busy with work. (#015: 27-year-old mother living with HIV; 2 children)*

Despite being highly motivated to attend well-care services, mothers described well-care visits becoming less frequent and punctual. There was a shift in priorities once children got older. For example, mothers may return to school or move away once the well-care visit schedule became less intensive. Nurses also stopped indicating exact dates for visits when children became older. Although there was no explicit intention to deprioritise well-care visit attendance, a shift in living arrangements and caregiving arrangements for children suggests that the role of mothers transitioned when children got older, which may have influenced attendance at well-care visits over time.

*I stopped taking him myself now that I am in Grade 12*,* my mother takes him. (#003: 18-year-old mother; 1 child)*

*I don’t miss [well-care visits] until she reaches 1 year. (#008: 23-year-old mother living with HIV; 3 children)*

ix. Adolescent mothers presenting themselves as “good moms” (TDF: Social/professional role and identity)

There was a reluctance among mothers to disclose experiences of missing well-care visits. Mothers were motivated to portray themselves as “responsible” and/or “good” mothers. This notion became clearer when contrasted against mothers’ opinions on the vignettes (i.e., hypothetical stories of a young mother struggling to attend health services). The use of vignettes solicited more nuanced reflections, where mothers felt more comfortable discussing the challenges of young and first-time motherhood when referring to other mothers. For example, mothers portrayed other young mothers as irresponsible because they neglected their children. This resulted in two opposing narratives; one where mothers portrayed themselves as responsible and caring for their children and the other portraying *other* mothers as irresponsible.

*I feel like I am not in the mood*,* but then I have no choice because I am a mother… I have to take this child… I have to cancel that job and not go to it because I have to take the child to the clinic… That job will pass and it will be given to someone else. (#014: 21-year-old mother; 2 children)*

On one hand mothers described themselves as having a sense of responsibility and making sacrifices for the benefit of their children’s needs. This extended beyond accessing child health services. For example, one mother spoke about delaying personal goals for their child’s immediate needs.

*You will delay many things. You will delay all those job opportunities*,* stay with the baby and have no space to do anything and concentrate on your child until being old. (#008: 23-year-old mother living with HIV; 3 children)*

This was juxtaposed by disapproving descriptions of other young mothers, emerging during dialogues about the vignette. Low attendance at health services and risk behaviours (e.g., abusing drugs/alcohol) were criticised and were blamed for children’s poor health.

*[My cousin] smoked pills during her pregnancy. Now her child has issues with asthma. His chest closes up… I think she didn’t go for her pre-natal check-ups. (#003: 18-year-old mother; 1 child)*

As a result of these contrasting descriptions of young mothers, mothers characterised their role and identity as young mothers as being centred around their children’s needs.

*I would tell her she needs to love her child and go all out for her child whether it’s bad or nice because it is not nice to raise a child especially in environments where we live in. (#011: 24-year-old mother living with HIV; 2 children)*

**Table 3:** Mapping of themes for well-care visit attendance behaviour on the integrated COM-B and TDF

COM-B	TDF	THEME*
CAPABILITY	Behavioural regulation	• Prioritising children’s health
		• **Indifference & tolerance of harsh attitudes from nurses**
	Knowledge	• Awareness of health promotion and prevention
		• Receiving guidance about well-care services & the RtH booklet
		• Good comprehension of well-care services and childhood vaccinations
	Memory, attention, & decision processes	• **Compliance with nurse’s schedule** Sub-theme: Decision to skip visits despite intention to adhere to schedule
	Skills	• **Organisational & financial acumen**
OPPORTUNITY	Environmental context & resources	• Child’s attendance is not influenced by adolescent mother’s availability
		• **Barriers: Barriers to access**,** lack of childcare support**,** service delivery factors**,** COVID-19**,** weather-related**
		• **Facilitators: RtH Booklet**,** Support**,** mother’s availability**,** accessibility of services**
	Social influences	• Child’s caretakers value attendance
MOTIVATION	Beliefs about capabilities	• Adolescent mother is responsible for taking child to clinic
		• **Opportunity to learn & obtain reassurance about child’s health**
	Beliefs about consequences	• **Fear of child illness if visits missed** Sub-theme: Avoid criticism from nurses when child is sick
		• Nurses penalise missed visits
	Goals	• Information seeking
	Intentions	• **Importance of well-care services & childhood immunisations** Sub-theme: Decline of well-care visit importance
		• Compliance with schedule
	Reinforcement	• Nurses monitor attendance
	Social professional role and identify	• Adolescent mother’s role is to care for child
		• **Adolescent mothers presenting themselves as “good moms”**

No themes were matched to the following domains of the TDF: emotions and optimism

^*^Major themes in bold

## Discussion

This study contributes to the limited evidence on well-care visit attendance among children born to adolescent mothers living in a rural and urban health district of the Eastern Cape, South Africa. Whilst attendance at the first scheduled visit was the lowest compared to all other visits, most children attended well-care services between 6 and 14 weeks. Overall, attendance declined through the first 18 months of life, with increases when visits coincided with the vaccination schedule. Qualitative analyses found that mothers were capable and motivated to attend visits. Despite the availability of kinship support to enable attendance, structural barriers to access (e.g. cost of transport and distance to the clinic) hindered children’s attendance.

### Patterns of attendance at well-care visits

As reported elsewhere [20], this study identified a critical gap in attendance during the neonatal period – a time of heightened risk for maternal and neonatal mortality, particularly among adolescent mothers [4, 57, 58]. Adolescent-specific barriers to postnatal care, including negative interactions with healthcare workers, psychological distress during the transition to motherhood, and perceptions that visits primarily benefit children rather than mothers, may be contributing to poor attendance [59, 60]. Further examination of vaccination and well-care visit data showed that coverage of birth vaccinations was high (≥ 95%), with most vaccines administered within 1–2 days after birth, while over a third of children attended the 3–6 day visit more than 7 days postpartum (Supplement 3). This suggests that children and their mothers may be receiving postnatal and newborn well-care alongside birth vaccinations at 1–2 days after birth rather than returning for a dedicated 3–6 day visit. Low attendance at the 3–6 day visit (48.7%) despite high vaccination coverage raises questions about the timing and added value of the this visit while highlighting the need to strengthen postnatal care uptake among children and their adolescent mothers.

Building on questions raised about the value of early well-care visits, records showed higher attendance when visits coincided with the vaccination schedule – for example, 51.6% at the 18-month visit compared to 20.2% at the 16-month visit. While interviews suggest that some mothers intentionally prioritised these visits [61], healthcare workers may be more likely to instruct children to return to visits when vaccinations are due, rather than for visits between vaccinations. Compared to WHO guidance, South Africa’s well-care visit schedule involves more frequent contact points in the first 18 months. An evaluation of the national well-care visit schedule may be warranted to assess whether possible efficiencies could be gained by optimising the timing and frequency of visits. Revising the schedule could reduce the burden of repeated clinic visits on adolescent mothers and alleviate pressure on healthcare workers by lowering patient load and waiting times.

Declining attendance from 85.1% at 6 weeks to 49.7% at 18 months highlights the need for interventions to support and improve retention in well-care services. This pattern mirrors age-related declines in vaccination coverage and follow-up visits among HIV-exposed infants of adolescent and older mothers in South Africa [24, 62–64]. Attendance declined with age despite mothers’ intention to adhere to the schedule as found in qualitative interviews. This may reflect diminishing perceived advantages of attending later visits as mothers’ need for information reduces and the necessity to return to education or work increases [65]. Waning attendance indicates that children at higher risk of low birthweight [2, 3, 7, 8] and undernutrition [9] may not be accessing services needed to promote healthy growth and prevent long-term consequences of being born with a low birthweight. By proxy, it also represents missed opportunities to deliver services to adolescents as recommended [1]. Nurses should maximise contact with adolescent mothers during their children’s well-care visits, to establish benefits to maternal and adolescent health. In turn, this may encourage mothers to continue taking children to visits themselves and enhance opportunities to reach adolescent girls. Increases in attendance at visit that coincide with the vaccination schedule, alongside mothers’ intention to adhere to the schedule, suggests that vaccination schedules may be an effective approach for reaching adolescent mothers and their children for health promotion and prevention interventions.

### Factors influencing well-care visits attendance

Qualitative analyses identified barriers and facilitators to well-care visit attendance, extending existing evidence on routine child health service attendance among children of adult caregivers [27, 39]. Motivations and strategies for ensuring attendance among mothers in this study were shaped by their understanding of the benefits of well-care for child health, their decision-making autonomy and maternal identities, and their ability to mobilise kinship support to overcome structural barriers.

Knowledge of the importance and benefits of vaccinations and growth monitoring for healthy child growth and development provided motivation for attendance. This contrasts with findings from Limpopo, where low awareness among adult caregivers was a barrier to attendance [27], and aligns with evidence from Nigeria [39]. The RtH booklet further facilitated understanding of the schedule and supported attendance, endorsing its recent re-design aimed at strengthening caregivers’ role in early childhood development [66, 67].

It was unexpected that harsh healthcare worker attitudes and long waiting times did not discourage attendance [16, 59, 68, 69]. Mothers’ decision-making autonomy and leading role in their children’s access to care also contrasted with evidence from KwaZulu-Natal, where adolescent mothers’ autonomy was constrained by age and financial dependence [70]. As also found among adolescent mothers across South Africa, mothers in this study prioritised visit attendance over their own comfort in the name of ‘good mothering’ and ‘doing the right thing’ [39, 40, 71–73]. Behaviours such as adhering to medical routines may represent actions undertaken by mothers to demonstrate their capacity to meet expectation of motherhood. Such behaviours have been described as “rituals of repair” by Moodley et al. to counter stigma and fractures in family relationships experienced during pregnancy [71]. Adopting mature, competent and responsible maternal identities during the transition to motherhood may help explain the decision-making autonomy and psychological resilience observed among mothers in this study [71, 72, 74, 75]. These processes may enable mothers to disregard stigmatisation and attenuate the influence of negative healthcare worker attitudes on attendance. Nevertheless, harsh treatment by healthcare workers remains commonly reported by adolescent mothers, including in this study [59, 72], underscoring the need for training healthcare workers to provide age-responsive, person-centred care that fosters compassionate and empathetic interactions with mothers.

As expected, mothers reported barriers to access such as financial, distance and transport barriers, long waiting times at facilities and service delivery disruptions due to COVID-19 pandemic [27, 49, 76]. However, as identified in South Africa and other low- and middle-income countries, this study demonstrates how kinship networks support children’s well-care visit attendance [59, 77]. Findings reveal the cash and in-kind support available to mothers in this study, including familial and intergenerational caregiving support [74, 78, 79]. The Eastern Cape has the highest proportion of children living with neither parent in South Africa [80], indicating a high level of reliance on kinship support networks for childcare. Research is needed to explore how kinship networks support attendance, especially considering that elderly caregivers, who may assume caregiving roles, can face unique access barriers such as restricted mobility. Altogether, the factors identified present favourable conditions that could be leveraged to support well-care visit attendance among children of adolescent mothers.

### Children of adolescent mothers living with HIV

In contrast to existing literature, our analysis of well-care visit records and qualitative interviews did not reveal differences by maternal HIV status [11, 12, 24]. Visit-specific differences observed by maternal HIV status in well-care visit records were exploratory and may reflect underlying age differences between groups. Qualitative findings and methodological limitations help contextualise why differential patterns by HIV status may not have emerged. First, mothers’ perception that visits mainly focused on growth monitoring and vaccinations may have constrained discussion of HIV-related care during well-care visits. This may have limited expression of HIV-specific experiences, potentially obscuring differences by maternal HIV status. Poor integration of HIV-related care within well-care services may have reinforced this perception [32]. Second, we expected that AMLHIV would report a higher reluctance to attend visits compared to HIV-negative adolescent mothers due to fears of early infant HIV diagnosis testing and stigma by healthcare workers [16, 81]. However, qualitative interviews revealed no notable differences by HIV status, with harsh treatment by healthcare workers reported across groups. This highlights the prevalence of poor healthcare worker attitudes towards mothers irrespective of HIV status. Thirdly, telephonic interviewing may have diminished the interviewer’s ability to establish necessary levels of rapport with AMLHIV to discuss challenges of securing their own and their child’s HIV-related care. During post-interview debrief meetings, we noted a reluctance among AMLHIV to elaborate on their experiences navigating PMTCT services alongside well-care services [79]. Further methodological limitations are described in the limitations section. Our findings highlight that healthcare-seeking behaviours are shaped by multiple and complex determinants. Maternal HIV status alone may not capture this complexity, particularly for adolescent girls navigating child health services following an unplanned pregnancy and early motherhood.

### Limitations

This study has several methodological limitations and strengths. Firstly, the absence of a comparison group of children of adult mothers prevents us from examining whether children of adolescent mothers have lower well-care visit attendance compared to children of older mothers. Secondly, since well-care visits are recorded manually, records are prone to recording error which could have led to an underestimation of well-care visit attendance. Furthermore, exclusion of children with missing or poor-quality RtH booklet data may have led to under- or overestimation of attendance; however, as the analysis focused on describing attendance patterns, descriptive findings of well-care visit attendance trends remain informative. Thirdly, the impact of remote interviewing on the quality of interviews has been debated [82, 83]. Our qualitative interviews may have been susceptible to recall bias due to the time delay between participants’ attending well-care as adolescent mothers and data collection, though the semi-structured interview guide was structured temporally, to help participants recall events. Societal pressures around ‘good mothering’ may also introduce reporting bias which was harder to remediate through establishing rapport due to telephonic interviewing. These potential sources of bias help us understand why narratives conveying a genuine intention and willingness to attend visits contradict with the decline in attendance observed in the well-care visit records. Lastly, this study did not include the perspectives of healthcare providers, limiting the ability to explore service delivery challenges that may influence the experiences of adolescent mothers and their children and affect attendance.

Despite these limitations, using a theory-informed approach facilitated a systematic exploration of factors that influenced well-care visit attendance among children and their adolescent mothers. The COM-B and TDF framework helped us isolate distinct areas where targeted intervention could support attendance. The longitudinal analysis of attendance over time provided unique insights into the trajectory of attendance patterns from birth to 18 months. Additionally, using a mixed-methods approach allowed us to elucidate the nuanced factors which may influence declining attendance over time.

## Conclusion

Children born to adolescent mothers are missing opportunities to receive health promotion and prevention services that support life-course health and well-being and reduce preventable child morbidity and mortality. Adolescent mothers’ motivations and strategies for ensuring well-care attendance present an already-existing scaffolding that health systems can leverage to reach adolescent girls and their children at heightened risk of adverse outcomes. Identifying opportunities to improve integration of well-care visits with HIV services may contribute to addressing suboptimal attendance identified in this study. Enhancing nurse-adolescent mother interactions and shifting from procedural care to developmentally appropriate care in line with WHO guidance could improve the perceived and actual value of well-care visits and encourage attendance. In turn, this may contribute to narrowing the health equity gap between children born to adolescent mothers and adult mothers.

## Supplementary Information

Supplementary Material 1.

Supplementary Material 2.

## Data Availability

Data are available on reasonable request - visit the study website for further information . Research materials are also available on the study website.
