# Supplementary material for: Well-care visit attendance among children of adolescent mothers in South Africa: a theory-informed mixed-methods study
Source: BMC Public Health. 2026 May 28;26:1714. doi: 10.1186/s12889-026-27326-z (PMC13217663; doi:10.1186/s12889-026-27326-z)
Supplement: Supplementary file 1 — Supplementary Material 1. [file 12889_2026_27326_MOESM1_ESM.docx]

**Supplementary Materials**

**Supplement 1: Proportion of children who attended well-care visits children among children ≥19 months (n=415) by maternal HIV status**

|  | Total (n=415) | Children of AMLHIV (n=166) | Children of HIV-negative adolescent mothers (n=249) | p-value |
| --- | --- | --- | --- | --- |
| 3-6 days | 48.7 (43.8-53.6) | 48.8 (41.2-56.4) | 48.6 (42.4-54.8) | 1.0 |
| 6 weeks | 85.1 (81.3-88.4) | 83.1 (77.4-88.8) | 86.3 (82.1-90.6) | 0.4 |
| 10 weeks | 81.2 (77.1-84.9) | 77.7 (71.4-84) | 83.5 (78.9-88.1) | 0.1 |
| 14 weeks | 73.7 (69.2-77.9) | 71.1 (64.2-78) | 75.5 (70.2-80.8) | 0.3 |
| 4 months | 45.8 (40.9-50.7) | 39.2 (31.7-46.6) | 50.2 (44-56.4) | 0.03 |
| 5 months | 43.4 (38.5-48.3) | 38 (30.6-45.3) | 47 (40.8-53.2) | 0.07 |
| 6 months | 64.6 (59.8-69.2) | 61.4 (54-68.8) | 66.7 (60.8-72.5) | 0.3 |
| 7 months | 38.1 (33.4-42.9) | 33.1 (26-40.3) | 41.4 (35.2-47.5) | 0.09 |
| 8 months | 33.7 (29.2-38.5) | 26.5 (19.8-33.2) | 38.6 (32.5-44.6) | 0.01 |
| 9 months | 61.2 (56.3-65.9) | 56 (48.5-63.6) | 64.7 (58.7-70.6) | 0.08 |
| 10 months | 30.6 (26.2-35.3) | 28.9 (22-35.8) | 31.7 (25.9-37.5) | 0.5 |
| 11 months | 25.8 (21.6-30.3) | 22.3 (16-28.6) | 28.1 (22.5-33.7) | 0.2 |
| 12 months | 62.2 (57.3-66.9) | 59.6 (52.2-67.1) | 63.9 (57.9-69.8) | 0.4 |
| 14 months | 32.5 (28-37.3) | 30.1 (23.1-37.1) | 34.1 (28.2-40) | 0.4 |
| 16 months | 20.2 (16.5-24.4) | 17.5 (11.7-23.2) | 22.1 (16.9-27.2) | 0.3 |
| 18 months | 51.6 (46.6-56.5) | 49.4 (41.8-57) | 53 (46.8-59.2) | 0.5 |

**Supplement 2: Summary of Participant Characteristics for Qualitative Interviews**

|  | Mother’s age at birth of eldest child (years) | Mother’s age at interview (years) | HIV status | Parity | Mother and child living together | Caregiver(s) involved in well-care visit attendance | Transport to  well-care visits |
| --- | --- | --- | --- | --- | --- | --- | --- |
| 1 | 18 | 26 | Negative | 2 | Yes | Mother and grandmother; child's father provides money for transport | Unknown |
| 2 | 20 | 24 | Negative | 2 | Yes | Mother | Hike/taxi and walk  (clinic far away) |
| 3 | 14 | 17 | Negative | 1 | No | Mother and now grandmother | Hike/taxi and walk  (clinic far away) |
| 4 | 16 | 22 | Negative | 3 | No | Mother and now grandmother | Unknown |
| 5 | 16 | 19 | Negative | 2 | Yes | Mother | 1h30 walk and bus |
| 6 | 18 | 23 | Negative | 2 | Yes | Mother, aunt and grandmother | 20-35 mins walk |
| 7 | 15 | 26 | Positive | 3 | Yes | Mother | 10 mins walk |
| 8 | 18 | 22 | Positive | 3 | Yes | Accompanied by Aunt or Mom | Taxi (Hike/taxi and walk (clinic nearby) |
| 9 | 19 | 23 | Positive | 2 | Yes | Mother | Hike/taxi  (10 mins; clinic nearby) |
| 10 | 18 | 23 | Positive | 2 | No | Mother and grandmother if at school | 1h30 taxi |
| 11 | 18 | 23 | Positive | 2 | Yes | Mother and grandmother if at work | Four taxis away |
| 12 | 18 | 23 | Positive | 2 | Yes | Mother | Walk (clinic nearby) |
| 13 | 14 | 18 | Negative | 1 | Yes | Mother and grandmother | Walk |
| 14 | 16 | 21 | Negative | 2 | No | Mother and now grandmother | Unknown (clinic nearby) |
| 15 | 19 | 27 | Positive | 2 | Yes | Mother and now aunt | 10 mins taxi/30 mins walk |
| 16 | 15 | 18 | Negative | 1 | Yes | Mother and grandmother | Unknown (clinic nearby) |

**Supplement 3: Comparison of age (in days) at recorded well-care visit and birth vaccinations among children ≥19 months (n=415)**

|  | Day 3-6 well-care visit  % (n) | BCG vaccination^1^  % (n) | OPV0 vaccination^1^  % (n) |
| --- | --- | --- | --- |
| Coverage (recorded date)*^2^* | 48.4 (200) | 96.1 (394) | 98.5 (404) |
| *Age at intervention^3^* |  |  |  |
| 0-2 days | 12.5 (25) | 81.5 (331) | 81.9 (331) |
| 3-6 days | 51.5 (103) | 11.7 (46) | 11.1 (45) |
| 7-14 days | 27.0 (54) | 3.6 (14) | 4.0 (16) |
| ≥15 days | 9.0 (18) | 3.3 (13) | 3.0 (12) |
| *^1^*Childhood vaccinations recommended at birth: Bacillus Calmette-Guérin (BCG) vaccine to prevent tuberculosis and the first dose of the Oral Polio Vaccine (OPV0).  *^2^*Proportion of children with a recorded date for each intervention.  *^3^*Age at intervention is calculated by subtracting the child’s date of birth from the recorded intervention date. Percentages reflect the distribution of intervention timing among children with a recorded date for that intervention. | | | |
