# Supplementary material for: Well-care visit attendance among children of adolescent mothers in South Africa: a theory-informed mixed-methods study
Source: BMC Public Health. 2026 May 28;26:1714. doi: 10.1186/s12889-026-27326-z (PMC13217663; doi:10.1186/s12889-026-27326-z)
Supplement: Supplementary file 2 — Supplementary Material 2. [file 12889_2026_27326_MOESM2_ESM.docx]

**Appendix 1: Interview Guide for Qualitative Interview**

**Introduction to interview**

We are interested in health services for mothers and their children. We also want to hear what you think about “well-care services”. These are the health services your child gets when they are “well” and not sick (example: vaccinations, weight measured). In the interview I will refer to these services as “well-care visits”.

Do you have any questions about what well-care visits are?

In this interview we are interested in hearing about your experiences with your youngest child. Please remember that everything you share will not link back to you. We want you to feel free to share your true experiences.

Do you have [child name] Road to Health booklet with you now?

We can take breaks whenever you need, please let me know.

There are no right or wrong answers – we are interested in hearing about **your** experiences.

If any questions do not make sense, please let me know and I will try to explain it more clearly.

| Required questions   - *Probes* |
| --- |

**SECTION 1: WARM UP**

Warm-up question 1: Can you tell me a bit about your children?

- *How old are they?*
- *Where do they live?*
- *Who takes care of them?*

Warm-up question 2: How old were you when you were last pregnant? How as your pregnancy?

- *How has your children’s health been since they were born?*
- *Can you tell me what kind of health services you and your child have had since you were pregnant with your child? When you did you register for antenatal care?*

**SECTION 2: ROAD TO HEALTH BOOKLET**

1. How would you describe what the Road to Health booklet to a new mother?

- *What pages would you tell her are the most useful/important?*

**SECTION 3: PLANNING (FREQUENCY & TIMING OF) WELL-CARE VISITS**

1. Can you tell me about taking your child to well-care visits?

- *Do you remember how the healthcare workers made you feel?*
- *Did you feel supported or would like more help? In what way?*

1. How do you plan usually well-care visits for your child?

- *Does anyone help you?*

| For any mother it can be really hard to learn everything about being a mother and to keep up with all appointments the well-care visits.  Every mother faces her own unique challenges. For some mothers it is difficult to have transport money while others have work or school obligations which make it hard for them to take her children to the clinic. |
| --- |

1. Can you think of any other reasons that it might be difficult for a young mother to take her child to the clinic?

- *What kind of advice would you give to her?*

1. Have any of those things ever happened to you?

- *What happened?*
- *Did you get any help?*
- *How did you solve it?*

1. Can you tell us a bit more about what you know about this schedule?

- *What do you think about this schedule?*
- *Do you keep track of the schedule?*

| Some mothers think it is very important to go to every clinic visit while others think it is less important. Sometimes it can feel like a waste of time to go to the well-care visit if you can see your child is growing well and they don’t need any vaccinations. |
| --- |

1. Can you think of any reasons why a young mother might decide not to take their child to the clinic?

- *What do you think would happen if she decided not to take her child?*
- *Can you remember a time when you decided not to take your child to the clinic?*
- *What happened?*

**SECTION 4: ATTENDING WELL-CARE VISITS**

| When Kholiswa became a mother for the first time, she was really excited about having a daughter, but she was also quite worried and stressed. There were a lot of things to plan and do for her baby. After her baby was born, she was given a Road to Health booklet and told to come to the clinic for check-ups every month until her baby was 12 months old. Sometimes Kholiswa was not able to take her daughter when she needed vaccinations and other times the clinic did not measure her daughter’s weight. |
| --- |

1. Can you tell me a bit about your last well-care visit?

- *Can you describe the process of what happened from beginning to end?*
- *How did you travel to the clinic?*
- *What happened once you arrived?*
- *What did the nurses do?*
- *Describe what happens with the book at the clinic.*
- *Were you given an appointment? Did you go on a specific day?*
- *Did you have any challenges?*
- *Can you describe what happened?*
- *How did you solve it?*

1. When you think about your child’s health visits, did something ever happen that you’ll never forget? What happened?
2. How did your most recent visit compare to others?
3. Have you ever been offered any family planning support or contraception during the well-care visits?

- *Can you tell me more about that? When does it happen? Who offers this?*
- *Did you seek this yourself or was it offered to you?*
- *How would you go about getting family planning services or contraception?*

*HIV tests & PMTCT services (only for mothers living with HIV)*

| When Kholiswa found out she was pregnant she had to do an HIV-test. This is when she found out about her HIV status. Since then, Kholiswa needs to take medication for her and her baby to stay healthy. After her daughter was born, her daughter also needed to take medication and get tested during check-up visits. Sometimes it was hard to make sure that her daughter received all the services she needed during a visit to the clinic. Kholiswa sometimes found it hard to know where she needed to go and who to ask questions to. Sometimes there wasn’t enough time during a visit to get all the services her daughter needed. |
| --- |

1. What do you think of Kholiswa’s story?

- *Have you ever had any similar experiences at the clinic?*

1. Can you tell me about the last time your child got an HIV test?

- *Can you describe the process of what happened from beginning to end?*
  1. *Did this happen during a well-care visit?*
  2. *Did you have to plan a separate visit?*
  3. *Do you know where to go (which clinic) to get HIV services for you and your child?*
- *Did you get any other HIV-related services?*
- *Did you experience any challenges?*
  1. *What happened?*
  2. *How did you solve it?*
  3. *Did you receive any support?*

1. Do you know when you are supposed to go for your child’s next HIV test?

*Mother’s health*

| We've talked a lot about your child's health, but the health of you as a mother is so important. I’d like to ask some questions about your health during the time when you needed to take your child to well-care visits. |
| --- |

1. How was **your** health during the time?
2. Do you feel like the nurses are also concerned about your health during the well-care visits?
3. Do you think there were any benefits for you going to the well-care visits?

- *Were the well-care visits useful for your health?*
- *I noticed in your child’s road to health booklet that you have a caesarean birth, were you ever asked about this during your child’s well-care visits?*
- *Did you receive the information that you need?*

1. What services or help do you think can help mothers like you stay healthy during pregnancy and while breastfeeding?

**SECTION 5: Experience at health facility, healthcare services improvements & preferences**

1. Have you ever been treated so badly by a healthcare worker that it made you nervous about going back to the same clinic? What happened?

- *How did that make you feel?*
- *Have you ever had a very positive experience with a healthcare worker? What happened? How did you they make you feel supported?*
- *Do you think nurses treat differently now that you are an older mother compared to when you were a younger mom?*

1. Could you describe your overall experience of the healthcare system?

- *How does it/ doesn’t it take into account your individual wishes and needs?*

1. Do you go to the same facility for your own health as you do for your child, or do you go to a different facility?

- *Can you share some reasons by you choose to go to this clinic?*
- *What influences the way you choose a clinic?*
- *Which health facility does your child go to for health visits?*
- *Do you always go to the same clinic?*
- *If not, why did you go to a different clinic?*

1. If you could change or improve one thing about health services for **your child**, what would it be?

**SECTION 6: CONCLUSION**

1. Is there anything else you would like to share with us before we finish?

Thank you for taking the time to answer our questions. We appreciate your time and honesty. We will use this information to tell government and organisations how to help young parents and children, like you.

Remember that you are an amazing young woman. Being a mother is not easy but is such an important job to do. And we are proud of you for all the care and love you give.

**Appendix 2: Reflection form for Qualitative Interview**

**QUALITATIVE INTERVIEW REFLECTION FORM**

*Form to collect reflections on the telephonic semi-structured interviews.*

| **Date completed:** | *DD/MM/YYYY* |
| --- | --- |
| **Name of person completing form:** | *NAME SURNAME* |
| **Reflection form code:** | *RF001/[G/B]/[Participant ID number]* |
| **Date activity conducted** | *20210301* |
| **Place conducted** | *Via cell phone* |
| **Time period** | *30:08 (minutes)* |

*Mark* ***X*** *in the sampling grid below based on participant’s characteristics*

|  | *Adolescent mother living with HIV* | *HIV-negative adolescent mother* |
| --- | --- | --- |
| *Primipara (one child)* |  |  |
| *Currently attending well-care visits* |  |  |
| *NOT currently attending well-care visits* |  |  |
|  |  |  |
| *Multiparous (multiple children)* |  |  |
| *Currently attending well-care visits* |  |  |
| *NOT currently attending well-care visits* |  |  |

*Use the spaces provided below to answer the bolded questions (non-bolded information is useful as prompts to assist in capturing detailed answers).*

| **Please provide a brief summary of the interview conducted.**   - Do you feel you were able to ask all the questions? - Describe setting and the people present (e.g., even if the baby was in the background) |
| --- |
|  |
| **Please write down your reflections about the call.**   - What is your general feeling/impression of the call? - How did the participant receive you?Did/do you have any thoughts about the call or the participant that surprised/surprises you? - How did the process of consent go? |
|  |
| **Please write down your reflections on the questions and probes.**   - How did participants respond? - What was difficult/went well? - Did the questions work? Where the probes helpful? - Where there any questions that you did not use? - PILOT: What did the participant think about the questions? Were there any suggested changes? |
|  |
| **Please write down your reflections the participant’s responses.**   - What questions were easy and what was difficult? How did you use to navigate those challenges? - Did the participant ask you any questions? - Did anything happen that you did not expect? |
|  |
| **What interesting things did you hear? Please write down any interesting quotes. You can paraphrase if you are not able to remember the exact wording.** |
|  |
| **Is there anything that is really important about this participant that we need to know?** |
|  |
| **Please write down anything you may have felt, thought, or experienced post-interview.** This can also include any feelings towards a situation, for example, if a participant speaks about a breakup or struggling with daily survival or COVID-19. Did you feel uncomfortable at any time? Did you learn anything from the experience? |
|  |
